# Polyadenylation ligation‐mediated sequencing (PALM‐Seq) characterizes cell‐free coding and non‐coding RNAs in human biofluids

**DOI:** 10.1002/ctm2.987

**Published:** 2022-07-20

**Authors:** Zhongzhen Liu, Taifu Wang, Xi Yang, Qing Zhou, Sujun Zhu, Juan Zeng, Haixiao Chen, Jinghua Sun, Liqiang Li, Jinjin Xu, Chunyu Geng, Xun Xu, Jian Wang, Huanming Yang, Shida Zhu, Fang Chen, Wen‐Jing Wang

**Affiliations:** ^1^ BGI‐Shenzhen Shenzhen China; ^2^ Obstetrics Department Shenzhen Maternity and Child Healthcare Hospital Shenzhen Guangdong Province China; ^3^ College of Life Sciences University of Chinese Academy of Sciences Beijing China

**Keywords:** cell‐free RNA, RNA sequencing, biofluids, in vitro diagnosis

## Abstract

**Background:**

Cell‐free messenger RNA (cf‐mRNA) and long non‐coding RNA (cf‐lncRNA) are becoming increasingly important in liquid biopsy by providing biomarkers for disease prediction, diagnosis and prognosis, but the simultaneous characterization of coding and non‐coding RNAs in human biofluids remains challenging.

**Methods:**

Here, we developed polyadenylation ligation‐mediated sequencing (PALM‐Seq), an RNA sequencing strategy employing treatment of RNA with T4 polynucleotide kinase to generate cell‐free RNA (cfRNA) fragments with 5′ phosphate and 3′ hydroxyl and RNase H to deplete abundant RNAs, achieving simultaneous quantification and characterization of cfRNAs.

**Results:**

Using PALM‐Seq, we successfully identified well‐known differentially abundant mRNA, lncRNA and microRNA in the blood plasma of pregnant women. We further characterized cfRNAs in blood plasma, saliva, urine, seminal plasma and amniotic fluid and found that the detected numbers of different RNA biotypes varied with body fluids. The profiles of cf‐mRNA reflected the function of originated tissues, and immune cells significantly contributed RNA to blood plasma and saliva. Short fragments (<50 nt) of mRNA and lncRNA were major in biofluids, whereas seminal plasma and amniotic fluid tended to retain long RNA. Body fluids showed distinct preferences of pyrimidine at the 3′ end and adenine at the 5′ end of cf‐mRNA and cf‐lncRNA, which were correlated with the proportions of short fragments.

**Conclusion:**

Together, PALM‐Seq enables a simultaneous characterization of cf‐mRNA and cf‐lncRNA, contributing to elucidating the biology and promoting the application of cfRNAs.

## Graphical abstract

PALM‐Seq simultaneously characterizes the abundance and molecular signature of cell‐free coding and non‐coding RNAs. The profiles and end motifs of cf‐mRNA and cf‐lncRNA can distinguish biofluids. Seminal plasma tends to retain longer fragments than other biofluids. Pyrimidine dominates at the 3′ ends of cf‐mRNA and cf‐lncRNA fragments, and its frequency is correlated with the fragment length.

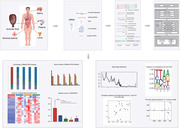

## 1 BACKGROUND

The existence of cell‐free RNA (cfRNA) in blood plasma was first recognized in the 1940s.^1^ Cell‐free messenger RNA (cf‐mRNA) and long non‐coding RNA (cf‐lncRNA) are released from tissues and carry rich information, making them among the most important components in liquid biopsy due to the easy accessibility.^2^ With the advancement of high‐throughput sequencing, cfRNA sequencing is becoming popular to investigate the abundance of cf‐mRNA and cf‐lncRNA instead of microarray in recent years, promoting the application of cfRNA. Maternal plasma cf‐mRNA and cf‐lncRNA, containing RNA derived from the placenta, reflect the longitudinal changes of pregnancy and could be used to identify pregnancies at risk of preterm birth and preeclampsia.^3^, ^4^, ^5^ cf‐mRNA transcripts in the blood plasma of cancer patients have the potential to predict the tumour origin, detect the cancer subtype and monitor the pathology.^6^, ^7^, ^8^ Plasma cf‐mRNA is also correlated with cognitive impairment in patients with Alzheimer's disease (AD),^9^ allowing the evaluation of disease‐related alterations in the brain. Besides, the observed pre‐symptomatic increase of a cf‐mRNA is associated with the development of AD.^10^ Thus, cfRNA shows advantages in the non‐invasive characterization of physiological and pathological conditions and provide promising biomarkers for monitoring, prediction, diagnosis and prognosis.

However, cfRNA is scarce and highly degraded, bringing a huge challenge to sequencing. Two major approaches have been applied to sequence cfRNAs, including RNA sequencing (RNA‐Seq) for low‐quality RNA samples and for small RNAs. Usually input of millilitres of plasma are required for RNA‐Seq library preparation, and some studies use capture kits of coding RNA to enrich cf‐mRNA.^3^, ^6^, ^7^ However, inconsistent results have been generated^11^, ^12^, ^13^, ^14^, ^15^, ^16^, ^17^, ^18^ and the performance on cf‐lncRNA is rarely evaluated.^19^ Treatment with T4 polynucleotide kinase (PNK) converts cfRNA to 5′ phosphate and 3′ hydroxyl and allows the library preparation using conventional ligation‐based small RNA‐Seq methods.^20^, ^21^ Such modified small RNA‐Seq methods have increased the detection of cf‐mRNA and cf‐lncRNA and can identify tissue‐specific RNA transcripts. Nevertheless, the low sequencing depth of cf‐mRNA and cf‐lncRNA is a common issue, mainly resulting from the large fraction of ribosomal RNA (rRNA), although different ranges of size selection have been carried out to improve the performance. These two approaches aim to capture long or short cfRNA fragments respectively and can both identify tissue‐specific transcripts, which are valuable to liquid biopsy. cfRNAs are usually released into surrounding body fluids through cell apoptosis, necrosis or exocytosis.^22^, ^23^ Cell‐free fragments of mRNA and lncRNA could be cleaved by ribonuclease A (RNase A) superfamily members,^24^ which are abundant in biofluids and prefer to cleave unpaired C and U at the 3′ end leaving 3′ phosphate and 5′ hydroxyl ends.^25^ The majority of cfRNAs are short RNA fragments (<200 nt), and long fragments (>200 nt) have been observed in blood plasma with various intensities due to different measurements.^6^, ^26^ The length distribution of cf‐mRNA and cf‐lncRNA remains largely unknown, as long RNA consists of mRNA, lncRNA, 18S and 28S rRNA and rRNA accounts for about 80% cellular RNA. Moreover, whether cf‐mRNA and cf‐lncRNA molecules carry the signatures of RNases still needs to be investigated. As the end motifs of cell‐free DNA fragments have been recognized as ‘fragmentomics’ markers in cancer, pregnancy, and transplantation,^27^ a novel method is demanded to characterize cf‐mRNA and cf‐lncRNA for better understanding the biology and application.

Therefore, we developed polyadenylation ligation‐mediated sequencing (PALM‐Seq) to capture original cf‐mRNA and cf‐lncRNA along with other informative RNA fragments. Through the treatment with T4 PNK and template‐independent addition of poly(A) tail, RNA fragments could be ligated to adaptors and reversely transcribed to complementary DNA (cDNA) with the removal of abundant RNA by the RNase H method. The sequencing of fragments regardless of length enables the analyses of the profile, fragment length and ends preference. We further applied PALM‐Seq to describe cfRNA in maternal blood plasma during pregnancy and profiled cfRNA in various biofluids. Our study provides an improved method to simultaneously characterize cf‐mRNA and cf‐lncRNA.

## 2 METHODS

### 2.1 Sample preparation and cfRNA extraction

We recruited healthy individuals and pregnant women to collect body fluids, including blood plasma, urine, saliva, seminal plasma and amniotic fluid. The information of participants is listed in Table S1. After fasting, blood samples were collected using K2 EDTA blood collection tube (BD, cat. 367863). Blood plasma samples for pooling plasma evaluation were pooled together before cfRNA extraction. Urine was collected in sterile cups. Saliva samples were collected by allowing passive drooling. Amniotic fluid was collected from four healthy pregnant women by amniocentesis. Seminal plasma samples were collected from four healthy males. All the biofluid samples were collected at Shenzhen Maternity and Child Healthcare Hospital. The study was approved by the Institutional Review Board of BGI‐Shenzhen (No. IRB20180420004). All subjects provided written consent forms prior to enrolment.

The fluid biopsies except seminal plasma were centrifuged for 10 min at 1600 *g* at 4°C. The upper layer liquid was collected carefully and then centrifuged for another 10 min at 14 000 *g* at 4°C. For seminal plasma, the spermatozoa were removed by centrifuging at 10 000 *g* for 30 min at 4°C. The supernatant of all biofluids was collected and mixed with TRIzol LS (Thermo Fisher Scientific, cat. 10296028) by vigorous shaking with a ratio of 1:3. For each library, 300 μl of plasma, 300 μl of amniotic fluid, 200 μl of seminal plasma, 300 μl of saliva and 1 ml of urine were used to extract cfRNAs following the manufacturer's manual. Five micrograms of RNase‐free glycogen (Thermo Fisher Scientific, cat. AM9510) was added as a carrier.

### 2.2 PALM‐Seq library preparation and sequencing

#### 2.2.1 T4 PNK treatment (PNKT) and poly(A) tailing

For cfRNAs, 10 μl of cfRNA was mixed with 4.5 μl of DEPC water (BBI, cat. D1005‐500 ml), 2 μl of 10× RNase H buffer, 2 μl of ATP (New England Biolabs, cat. P0756), .5 μl of T4 PNK (New England Biolabs, cat. M0201, for no‐PNKT, DEPC water was used instead), .5 μl of poly(A) polymerase (PAP, New England Biolabs, cat. M0276) and .5 μl of an RNase inhibitor (Enzymatics, cat. Y9240L) and then incubated at 37°C for 30 min. For Universal Human RNA Reference (UHRR) or Human Brain Reference RNA (HBR), total RNA was mixed with DEPC water, 10× RNase H buffer and ATP to 18.5 μl, incubated at 94°C for 5 min to fracture the long RNAs and then mixed with T4 PNK, PAP and RNase inhibitor and incubated at 37°C for 30 min. For UHRR‐RNase A treatment, .2‐ng RNase A (BBI, cat. 0675‐100 mg) was added to 100‐ng UHRR, digested at 37°C for 15 min then was extracted by precipitation by adding an equal volume of iso‐propanol. Incubation at 94°C was omitted. For the External RNA Controls Consortium (ERCC) assay, 1 μl of 1:100 ERCC RNA Spike‐In Mix (Thermo Fisher Scientific, cat. 4456740) was added to the UHRR total RNA. A volume of 2‐μl stop‐annealing buffer (40‐mM EDTA, 1.5‐M KCl) was added to stop the reaction.

#### 2.2.2 5′ Adaptor ligation

A volume of 1‐μl depletion oligo mix composed of .5 μM of each oligo was added. Y RNA and vault RNA (vtRNA) depletion oligos are shown in Table S2, and rRNA depletion oligos were the same as previously reported.^28^ For no targeted RNA depletion (TD) treatment, DEPC water was used instead. The mixture was incubated at 95°C for 5 min, and the temperature was decreased to 22°C at a rate of .1°C/s. Then the cfRNA was placed on ice.

A volume of 4‐μl 10× T4 RNA ligase buffer, 4 μl of ATP, 7 μl of 50% PEG, 1 μl of T4 RNA ligase 1 (New England Biolabs, cat. M0204), .5 μl of RNase inhibitor and .5 μl of 20‐μM 5′ RNA adaptor were added. The samples were then incubated at 25°C for 1 h. Then 2 μl of rSAP (New England Biolabs, cat. M0371) was added and incubated at 37°C for 10 min to remove ATP.

#### 2.2.3 Abundant RNA depletion

A volume of 1‐μl RNase H (New England Biolabs, cat. M0297) together with 5 μl of DEPC treated water was added (in the case of no TD treatment, DEPC water was used instead) and incubated at 37°C for 30 min (in the case of no TD treatment, this step was skipped). Then 2 μl of DNase I (New England Biolabs, cat. M0303) together with 3 μl of 30‐mM CaCl_2_ was added and incubated at 37°C for 30 min to remove the depletion probe and residual cell‐free DNA. A volume of 50‐μl RNA Clean Beads (Vazyme, cat. N412) were used to purify the modified RNA, and the RNA was resuspended in 12.5 μl of DEPC‐treated water.

#### 2.2.4 Reverse transcription (RT)

A volume of 1‐μl 5‐μM reverse transcription (RT) primer was added, and the mixture was incubated at 65°C for 5 min and then placed on ice for 2 min. A volume of 1‐μl 10‐mM dNTP, .5 μl of RNase inhibitor, 4 μl of 5× HiScript II buffer and 1 μl of HiScript II reverse transcriptase (Vazyme, cat. R201) were added and incubated at 50°C for 15 min, and the mixture was then incubated at 85°C for 5 min to inactivate reverse transcriptase.

#### 2.2.5 PCR amplification

A volume of 5‐μl PCR primers (5‐μM each) and 25 μl of 2× Q5 Hot Start PCR Master Mix (New England Biolabs, cat. M0494) were added. PCR was carried out following the manufacturer's manual, with annealing temperature of 56°C, extension time of 1 min and 20 cycles of amplification. The PCR products were purified with 75 μl of DNA Clean Beads (Vazyme, cat. N411) and then circularized.

#### 2.2.6 Sequencing

Sequencing was performed on BGISEQ‐500RS or MGISEQ‐2000RS to generate 100‐bp single‐end reads. In addition, 100‐bp paired‐end reads were generated in biofluids to evaluate the length of cfRNA fragments. The adaptors and primers are shown in Table S2.

Small RNA libraries were constructed by using an MGIEasy Small RNA Library Prep Kit (MGI, 1000005269) according to the kit's protocol. An amount of 10‐ng HBR was added to each library.

### 2.3 Data processing

Adaptors and poly(A) sequences were removed using cutadapt (v1.18)^29^ by the following multiple steps: (1) trimming 3′ sequencing adaptor; (2) trimming the low‐quality ends (sequencing quality <15); (3) removing reads that are shorter than 17 bp after adaptor trimming and quality trimming; (4) removing reads containing >10% of unknown bases N. After filtering, the clean reads were mapped to the rRNA, vtRNA and YRNA reference of human sapiens in NCBI using Bowtie (v1.2.2).^30^ For ERCC assay, we mapped the full set of reads to the set of ERCC transcripts. Then the remaining reads were aligned to multiple RNA references, followed by microRNA (miRNA, from miRbase^31^), other small RNAs (including transfer RNA [tRNA] from gtRNAdb^32^ and Piwi‐interacting RNA [piRNA] from piRBase^33^), mRNA and lncRNA (from GENCODE^34^) and other RNA biotypes in GENCODE, including Mt_rRNA, Mt_tRNA, misc_RNA, scRNA, snRNA, snoRNA, ribozyme, sRNA and scaRNA. We used Bowtie to perform the alignment with parameters: ‐v 1 ‐a –best –strata –norc. A similar mapping strategy was also recommended by the exceRpt pipeline.^35^

### 2.4 Quantification of the abundance of RNA

We generated miRNA reference using the name of mature miRNAs and the sequences of hairpin miRNAs. All reads assigned to a given miRNA were checked by comparing the mapping position with a known mature miRNA position in miRNA precursor, and the mapped reads that had an edit distance greater than 3 bp at the start or greater than 5 bp at the end were excluded. Then a custom Perl script was used to count the reads. To make them comparable, we normalized the RNA profiles to the read counts of a target RNA per million mapped reads (RPM). For tRNAs and piRNAs, we performed parallel alignment and allowed the reads to align across different biotypes simultaneously if they shared the same best mapping quality. piRNAs usually had a lower priority than tRNAs to ensure the correct assignment of reads, because the confidence of their annotations was lower. Finally, a custom Perl script was used to count the reads of various small RNAs and scaled to reads of a target RNA per million. For mRNA and lncRNA, the software RSEM (v1.2.9)^36^ was used to estimate the value of transcripts per million (TPM) as abundance of genes. Differentially abundant genes between samples were analysed using edgeR from R package TCC^37^ with the cut off *q* < .01, |log_2_FC| > 1. Differentially abundant genes in a specific biofluid were obtained by comparing samples belonging to that biofluid against to the rest of the samples (e.g. plasma vs. other biofluids). Differentially abundant genes during pregnancy were identified by comparing samples from first, second and third trimesters with non‐pregnant samples, with a cut‐off *q* < .05. The number of reads mapping to each ERCC transcript was counted using the samtools idxstats package^38^ and the count number was used for further analysis.

### 2.5 Profile and annotation

Functional annotation of enriched gene ontology (GO) and Kyoto Encyclopedia of Genes and Genomes (KEGG) pathway was performed by Metascape (https://metascape.org/gp/index.html).^39^ The tissue‐specific pattern genes were annotated according to the records of PaGenBase (http://bioinf.xmu.edu.cn/PaGenBase/). We used CIBERSORT and xCell to calculate the immune scores of five body fluids using the abundance matrix with TPM of mRNAs. For CIBERSORT, we run the R code (https://rdrr.io/github/IOBR/IOBR/man/CIBERSORT.html) with the ‘absolute’ mode to perform analysis and the output ‘absolute score’ for further analysis. The ‘ImmunoScore’ of xCell was analysed on the official online website (https://xcell.ucsf.edu) with default parameters.

### 2.6 Reverse transcription‐quantitative PCR (RT‐qPCR)

We conducted real‐time PCR following the polyadenylation‐based miRNA qPCR protocol, with a slight modification, that is, T4 PNK was added, whereas samples were treated with PAP. SYBR Green‐based qPCR was performed on the ABI StepOnePlus system using AceQ qPCR SYBR Green Master Mix (Vazyme, cat. Q141). The RT primers and qPCR primers are shown in Table S2.

### 2.7 Characterization of cfRNA fragments

We extracted the length of mapped reads from aligned bam files to count the fragment length for all types of RNAs. For paired‐end data, we first assembled paired reads and then analysed the merged reads as single‐end using our pipeline. Similarly, the length of the fragment was extracted from alignment files. Next, to evaluate the length of RNAs with insert fragment between sequencing reads, the paired reads were analysed in single‐end mode separately. If the first read had a mapping length of 90 bp, its paired read that mapped to the same transcript was kept. Then, the length of this fragment was calculated as the distance between the mapping loci of the paired reads plus the mapping length of the second read. Finally, we merged the count of each length in the previous two strategies and assigned as the number of fragments with certain length.

To reveal the molecular features of cfRNA fragments, we identified the locus that one base before the alignment locus to RNA reference as the 3′ end of mRNA or lncRNA fragments and then calculated the frequency of each type of nucleotide. The mapping locus of RNA reference was identified as 5′ end and the nucleotide was recorded for each fragment. For the 4‐mer end motif, we extracted the nucleotides from minus and plus two sites of the origin mapping region to RNA references. The frequency of nucleotide on each site and all 256 kinds of 4‐mer end motifs was counted and normalized to the value of percentage.

### 2.8 Statistical analysis

All statistical analyses were performed using R and Python. Principal component analysis (PCA) was calculated using the R package ‘prcomp’ function. Wilcoxon rank‐sum test was used to compare the ratio of sense and anti‐sense fragments. Differences between the preference nucleotides of 3′ end or 5′ end were analysed by matched Student's *t* test (two‐tailed). Two‐tailed Student's *t* test was also used to evaluate the differences between the absolute score of immune cells. The level of significance was identified as following: not significant, *p < .05, **p < .01, ***p < .001.

## 3 RESULTS

### 3.1 Design of PALM‐Seq for cf‐mRNA and cf‐lncRNA sequencing

**FIGURE 1:**
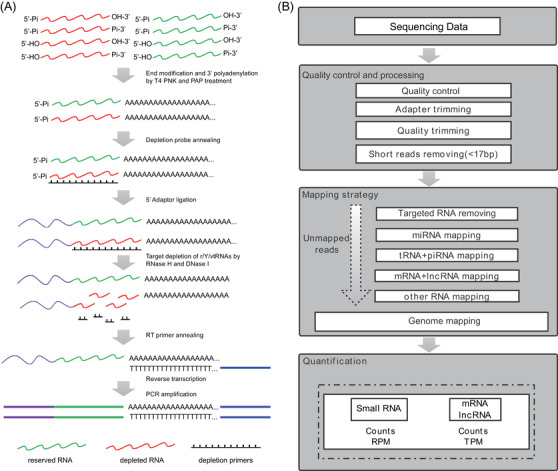
**Schema of polyadenylation ligation‐mediated sequencing (PALM‐Seq) library preparation and data analysis workflow**. (A) Experiment design plan. The library preparation process includes polynucleotide kinase (PNK) treatment and targeted depletion of abundant RNAs. (B) Data analysis workflow. Small RNAs are quantified by counts and reads per million (RPM), whereas mRNAs or long non‐coding RNAs (lncRNAs) are quantified by counts and transcripts per million (TPM)

As cf‐mRNA and cf‐lncRNA are highly degraded, with low concentration and various modified ends, we used T4 PNK to generate linear cfRNAs with 5′ phosphate and 3′ hydroxyl.^20^, ^21^, ^40^, ^41^ At the same time, PAP was added to polyadenylate the 3′ end of cfRNAs.^42^ Then, 5′ RNA adaptor was ligated by T4 RNA ligase 1.^43^ TD with RNase H and DNase I was designed to remove abundant rRNA, vtRNA and Y RNA. In contrast to 3′ DNA adaptor used in common small RNA‐Seq, 3′ polyadenylation and 5′ RNA adaptor could keep intact in the following RNase H‐based targeted RNA depletion^28^ and make it possible to eliminate abundant RNA and prevent DNA contamination. Finally, cfRNAs with 3′ polyadenylation and 5′ adaptor were reversely transcribed to cDNAs using oligo(dT) as an RT primer and the cDNAs were amplified by PCR for further preparation of libraries (Figure 1A).

The sequencing data of PALM‐Seq libraries underwent a highly stringent bioinformatic pipeline. Generally, the adaptors and poly(A) sequences were trimmed from the end of reads, and only reads longer than 17 bp were retained. The reads mapped to rRNA, vtRNA and Y RNA were then filtered. Next, an iterative alignment strategy was used to map the remaining reads to the RNA references, including miRNA, tRNA, piRNA, mRNA, lncRNA and other non‐coding RNAs (Figure 1B).

### 3.2 PALM‐Seq effectively captures cf‐mRNA and cf‐lncRNA

**FIGURE 2:**
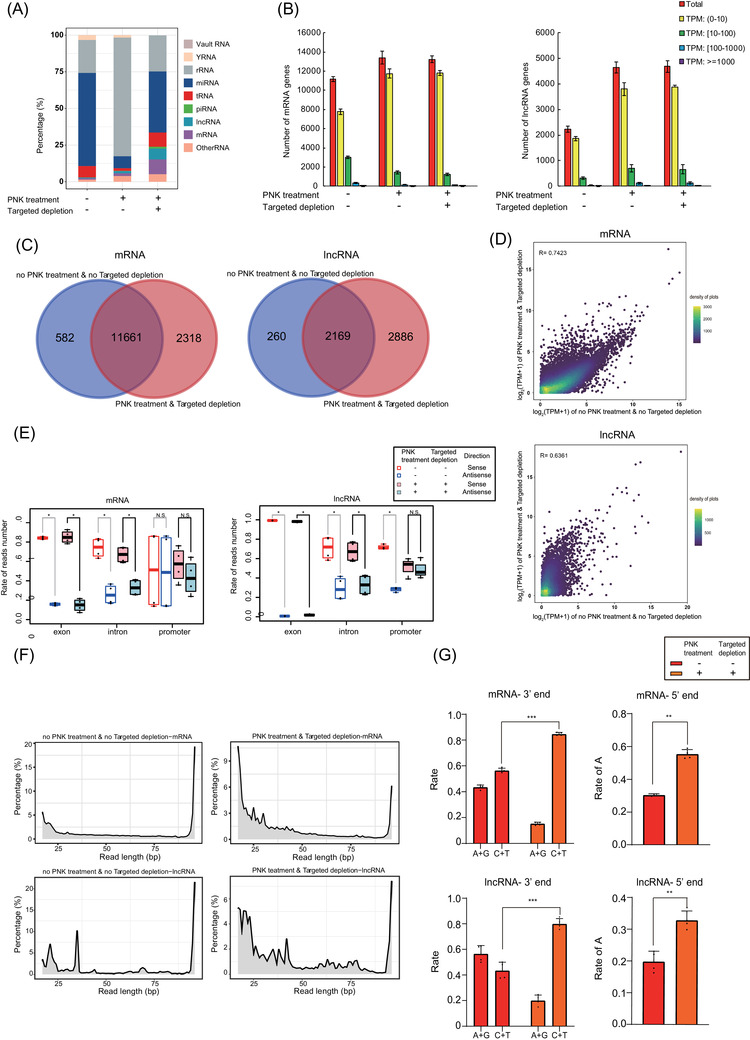
**Biotype distribution and complexity of RNA in different treatments of polyadenylation ligation‐mediated sequencing (PALM‐Seq)**. (A) The percentage of mapped reads of different RNA biotypes obtained by different treatments. (B) The number of mRNAs (left panel) and long non‐coding RNAs (lncRNAs) (right panel) detected by different treatments. (C) Venn diagram shows the number of detected mRNAs (left) and lncRNAs (right) by different treatments. Genes that could be detected in two samples or more are considered to exist in this group. (D) Profiles of mRNA (upper) and lncRNA (lower) with and without T4 polynucleotide kinase (PNK) treatment and targeted RNA depletion. The Pearson correlation is calculated through simple linear regression of log_2_(TPM + 1). (E) Boxplots that show the percentage of reads that locate on the sense and antisense strand. The reads were recognized as exons, introns or promoters on mRNAs or lncRNAs. Wilcoxon sum‐rank test is used to identify the significance with *p* < .001. (F) The distribution of fragment length of mRNAs (upper panel) and lncRNAs (lower panel) by different treatments. The *X*‐axis shows the different reads length (bp), whereas the *Y*‐axis shows the rate of each different size of sequence fragment. (G) The ratio of pyrimidine versus purine at the 3′ ends and the rate of adenine at the 5′ end of mRNAs (upper) and lncRNAs (lower) in PALM‐Seq libraries. The *p* value was calculated by matched Student's *t* test (two‐tailed), and the level of significant was identified as following: not significant (N.S.), *p < .05, **p < .01, ***p < .001

In order to verify the PALM‐Seq strategy for cf‐mRNA and cf‐lncRNA sequencing, we compared libraries with or without PNKT and TD of abundant RNA. We isolated cfRNA from blood plasma and constructed libraries, using 300‐μl plasma for each library. As expected, PNKT alters the frequency of various classes of RNAs. Compared to no_PNKT‐no_TD libraries, PNKT without TD increased the proportions of mRNA (1.9% vs. 1.0%) and lncRNA (1.4% vs. .5%) and r/Y/vt RNA (82.6% vs. 25.8%), as well as the number of detected mRNA (13403 vs. 11178) and lncRNA (4641 vs. 2233) (Figure 2A,B). The application of TD significantly reduced the rate of abundant RNA biotypes from 82.6% to 24.6% (Figure 2A). Furthermore, PNKT‐TD could detect more mRNA and lncRNA genes covering the majority of mRNA (11661 in 12243) and lncRNA (2169 in 2429) detected by no_PNKT‐no_TD, and they had a high correlation coefficient of abundance profile (Figure 2C,D, Table S3). The relatively low TPM values for most RNAs in the samples with treatments may be due to the increased number of detected genes. Adding asymmetric adaptors to both ends of cfRNAs by PALM‐Seq makes it extract the strand‐specific cfRNA fragments. The enrichment of sense orientation of mRNA and lncRNA fragments in both PNKT‐TD and no_PNKT‐no_TD groups revealed the strand‐specific feature of this method, as well as the elimination of DNA contamination (Figure 2E). These results confirmed that PNKT‐TD was suitable to profile cf‐mRNA and cf‐lncRNA in plasma.

Due to the lack of cfRNA standards, we used UHRR, a cell‐derived RNA standard for RNA‐Seq experiment,^44^ with ERCC RNA mix spiked, to evaluate the accuracy and sensitivity of RNA abundance quantification of PALM‐Seq. The correlation coefficient of ERCC indicated that PALM‐Seq had a high degree of similarity (*R* ^2^ > .94, Figure S1A). We successfully constructed libraries using 1‐, 5‐ and 10‐ng UHRR, respectively. When the input of UHRR reached 5 ng, the *R* ^2^ value of mRNA between technical replicates was .99 and the correlation coefficient between PALM‐Seq and reverse transcription‐quantitative PCR (RT‐qPCR) was comparable (Figure S1B). It demonstrated that PALM‐Seq enabled an accurate quantification of mRNA abundance with a low input RNA.

Moreover, the concentration of cfRNA in blood plasma varies from <1 to 5 ng/ml.^3^ To evaluate the required amount of input for this method, we performed PALM‐Seq on pooled blood plasma of 100 or 500 μl. For mRNA, the *R* ^2^ between three technical replicates of 100 μl of blood plasma was .92, and the value of 500 μl of blood plasma was above .97 (Figure S1C). A similar trend was observed in lncRNA (Figure S1D). The proportions and gene numbers of mRNA and lncRNA were similar between these two inputs (Figure S1E,F). The results proved that PALM‐Seq could profile cfRNAs from as low as 100 μl of blood plasma.

### 3.3 PALM‐Seq effectively captures cell‐free small RNAs

In order to retain informative RNA in biofluids, we did not carry out size selection or exome capture. It can be inferred that small RNAs should also be effectively captured in our method. 5′‐Phosphate containing small RNA fragments dropped out, whereas rRNA fragments increased. Small RNAs, such as miRNA, tRNA and piRNA, were quantified and compared between the treatment of no_PNKT‐no_TD and PNKT‐TD. Under PNKT condition, the number of detected miRNAs was slightly less than no_PNKT condition (Figure S2A), whereas the majority of detected miRNAs were shared by these two conditions and had a strong correlation of abundance (Figures S3A and S4). Meanwhile, PNKT could increase the proportions and detected gene numbers of piRNA and was with no significant influence on tRNA (Figures S2B,C and S3B,C).

To evaluate the accuracy and sensitivity of small RNA quantification, we presented the performance of PALM‐Seq on HBR, a standard for small RNA‐Seq experiment.^45^ We used different concentrations of HBR to construct the libraries. When the amount of input RNA reached 5 ng or more, the *R* ^2^ between technical replicates was above .94. We also constructed small RNA‐Seq libraries using 10‐ng HBR as an input, and the *R* ^2^ between PALM‐Seq and small RNA‐Seq was larger than .85, also confirming the consistency of PALM‐Seq (Figure S5A).

We further evaluated the sensitivity and accuracy of PALM‐Seq on plasma cell‐free small RNAs. For 100‐ and 500‐μl input volumes of blood plasma, the correlation coefficient of miRNA abundance was higher than .99, and the number of detected miRNA was similar (Figure S5B,E). The number of detected tRNA kept consistent for 100 and 500 μl of blood plasma inputs, whereas a larger amount of inputs increased the detection number of piRNA (Figure S5C,D,F,G). These results demonstrated that PALM‐Seq could profile small RNAs simultaneously with mRNA and lncRNA.

### 3.4 Characterization of RNA fragments by PALM‐Seq

As PALM‐Seq captures original RNA fragments in blood plasma, the molecular features of cfRNA including length and end bases are expected to be preserved. We tried to characterize RNA fragments captured by PALM‐Seq. We evaluated the length distribution of different RNA biotypes of UHRR libraries. Most reads from mRNA and lncRNA were longer than 90 bp (the upper limit of single‐end 100‐bp read) as expected, whereas the length distribution of miRNA and piRNA formed peaks between 20 and 40 nt (Figure S6). We then characterized the length distribution of cf‐mRNA and cf‐lncRNA under the PNKT condition. When PNKT was applied, more small fragments (<50 nt) of mRNA and lncRNA were captured (Figure 2F), which also implied that PNKT recruited degraded RNA fragments in biofluids. As RNases have preferential cleavage sites, we further calculated the rate of the nucleotides at the 3′ and 5′ ends of mRNA and lncRNA fragments. We found that the rate of pyrimidine (C and T) was significantly higher than that of purine (A and G) under PNKT conditions at the 3′ ends, and a preference of adenine at 5′ ends. However, in the absence of PNKT, the rates of pyrimidine and purine at 3′ ends were relatively equal, and the rate of adenine at 5′ ends was lower (Figure 2G). This feature was consistent with the cleavage preference of RNase A at the 3′ end of unpaired U and C residues and adenine at 5′ ends.^25^ It seemed that the treatment of T4 PNK largely recruited mRNA and lncRNA fragments degraded by RNase A. To exclude the probability of consequence of technical bias, we analysed the libraries constructed by phospho‐RNA‐seq,^20^ for which the RNA isolation method and library construction strategy were both different from ours. The preference of pyrimidine at the 3′ ends and adenine at 5′ ends (except for mRNA) of phospho‐RNA‐seq supported the recovery of RNase A–degraded RNA by T4 PNK (Figure S7). All the results showed that PALM‐Seq could capture mRNA and lncRNA fragments regardless of length, simultaneously detect small RNAs and characterize molecular signatures of RNA fragments.

### 3.5 Description of the change of blood plasma cfRNAs during pregnancy

**FIGURE 3:**
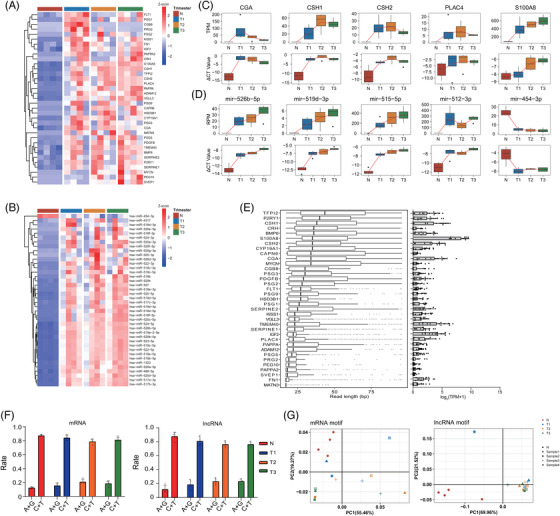
**Characterization of plasma cell‐free RNA (cfRNA) from pregnant women**. (A) Heat map of abundance of placenta‐specific genes, overlapped with differentially abundant cell‐free messenger RNA (cf‐mRNA) and long non‐coding RNA (cf‐lncRNA) between non‐pregnant and pregnant females at different trimesters. The data were converted to log_2_(TPM + 1) then scaled and clustered by rows. (B) Heat map of abundance of differentially abundant microRNA (miRNA). The data were converted to log_2_(RPM + 1) then scaled by rows. (C) Abundance of selected mRNA in polyadenylation ligation‐mediated sequencing (PALM‐Seq) (transcripts per million [TPM]) and reverse transcription‐quantitative PCR (RT‐qPCR) (ΔCT, normalized to B2M). (D) Abundance of selected miRNA in PALM‐Seq (RPM) and RT‐qPCR (ΔCT, normalized to RN7SL). (E) Boxplots (left) summarize the read length distributions for representative placenta‐specific mRNA and lncRNA across four pregnant females at three time points. Boxes represent the IQR, and whiskers represent first/third quartile 1.5*IQR. The right panel is the log_2_ (TPM + 1) of corresponding genes. (F) The ratio of pyrimidine and purine at the 3′ ends of mRNAs (left) and lncRNAs (right) in non‐pregnant and pregnant women at three time points. N: Non‐pregnant; T1: trimester 1; T2: trimester 2; T3: trimester 3. (G) Principal component analysis (PCA) plot of the frequency of 4‐mer end motifs of mRNA (left panel) and lncRNA (right panel). Each point represents individual non‐pregnant or pregnant women at three time points

Having confirmed that PALM‐Seq could capture mRNA, lncRNA and small RNAs, we used this method to evaluate the abundance and the molecular signatures of cfRNAs in blood plasma with the development of pregnancy. We performed PALM‐Seq on 16 blood plasma samples from 4 non‐pregnant and 4 pregnant women at 3 time points (12, 24 and 36 gestational weeks) (Table S3). Differentially abundant cf‐mRNA, lncRNA and miRNA were analysed between pregnant and non‐pregnant women, and a group of mRNA, lncRNA and miRNA genes were identified (Table S4), many of which are placenta‐specific (Figure 3A,B). Compared to the non‐pregnant women, the abundance of placenta‐specific protein‐coding genes *CGA*, *CSH1* and *CSH2* and the long non‐coding gene *PLAC4* ^4^ increased significantly during pregnancy. *S100A8*, which is related to pregnant immunomodulation,^4^ was increased significantly in the third trimester (Figure 3C). For example, *CGA* increased from .58 in non‐pregnant women to 75.5 in the first trimester (*q* < .001), 28.2 in the second trimester (*q* < .001) and 14.2 in the third trimester (*q* < .01). Placenta‐specific miRNA genes from the chromosome 19 miRNA cluster (C19MC),^46^ such as mir‐526b‐5p, mir‐519d‐3p, mir‐515‐5p and mir‐512‐3p, were with a very low amount or undetectable in plasma of non‐pregnant women and increased significantly during pregnancy. For example, the abundance of mir‐526b‐5p increased to 21.6 in first trimester (*q* < .001), 23.9 in the second trimester (*q* < .001) and 36.6 in the third trimester (*q* < .001) (Figure 3D). We verified five mRNA and lncRNA genes and five miRNAs using RT‐qPCR (Figure 3C,D). The identification of reported pregnancy‐related genes and their similarity with the results of RT‐qPCR indicated that PALM‐Seq successfully identified and accurately quantified pregnancy‐related cfRNAs in maternal blood plasma.

During pregnancy, the placenta releases nucleic acids into the blood,^4^, ^19^, ^47^ which may affect the composition of cfRNAs. Using PALM‐Seq, we could characterize the length distributions of the differentially abundant cf‐mRNA and cf‐lncRNAs. We found that most of the detected placenta‐specific genes had a large number of short fragments, ranging from 20 to 60 nt (Figure 3E). We investigated the rate of pyrimidine at the 3′ ends of cf‐mRNA and cf‐lncRNA fragments, and no significant change was identified (Figure 3F). The PCA of cf‐mRNA and cf‐lncRNA 4‐mer end motifs showed that non‐pregnant samples clustered in the left, apart from pregnant samples, and samples from the same pregnant woman collected at different trimesters tended to cluster together (Figure 3G), indicating that cfRNAs from plasma of pregnant women have unique characters. Our results demonstrated that maternal cfRNA carrying information of pregnancy presented on not only profiles but also molecular signatures.

### 3.6 Profiling of cfRNAs in biofluids

**FIGURE 4:**
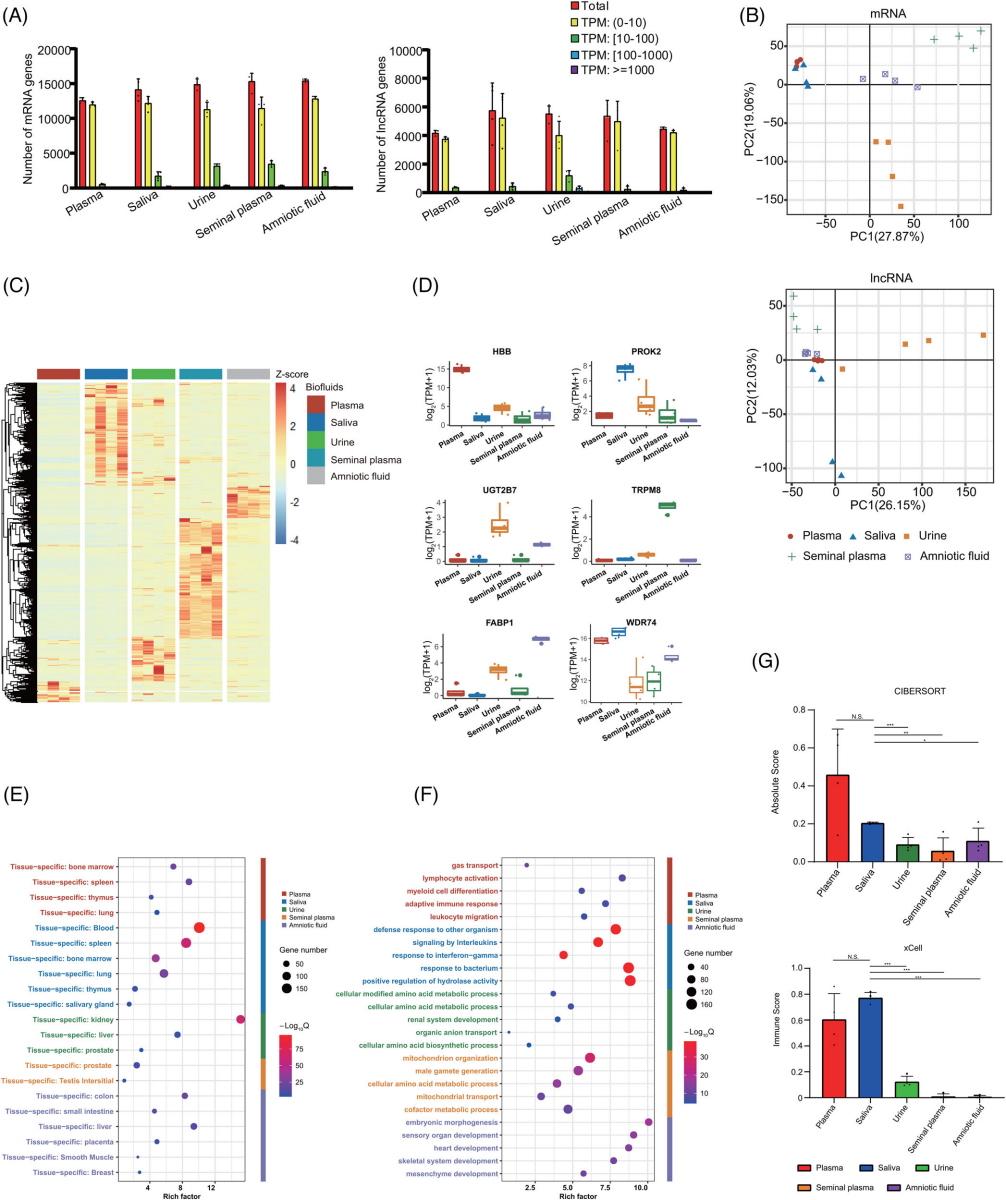
**Characterization of cell‐free RNAs (cfRNAs) of different biofluids**. (A) Number of mRNAs and microRNAs (miRNAs) detected in different biofluids. (B) Principal component analysis (PCA) plot using transcripts per million (TPM) of mRNA (upper) and long non‐coding RNA (lncRNA) (lower) TPM. Each point represents a single biofluid sample. (C) Heat map of differentially abundant genes in five biofluids (left panel). The data were converted to log_2_(TPM + 1) and then scaled and clustered by rows. (D) Abundance of representative genes in different biofluids. *X*‐axis represents different biofluids. *Y*‐axis represents values of log_2_(TPM + 1). (E) Plot of the origin of the enriched genes in different biofluid. *Y*‐axis represents specific tissues; *X*‐axis represents the ‘Rich Factor’, which means the percentage of all the user‐provided genes that are found in the given ontology term. (F) Plot of the enriched gene ontology (GO) biological process and Kyoto Encyclopedia of Genes and Genomes (KEGG) pathways of biofluid‐specific mRNAs. *Y*‐axis represents the pathways; *X*‐axis represents the ‘Rich Factor’. (G) The immune scores calculated by CIBERSORT (upper panel) and xCell (lower panel) in all five body fluids. Two‐tailed Student's *t* test was used to evaluated *p* value and the level of significant was identified as following: not significant (N.S.), *p < .05, **p < .01, ***p < .001

It is known that various biofluids contain cfRNAs.^48^ We next evaluated whether PALM‐Seq could profile cfRNAs in other biofluids. We applied PALM‐Seq to characterize cfRNAs in blood plasma, saliva, urine, seminal plasma and amniotic fluid from healthy individuals. The detected gene number of RNA biotypes was variable among biofluids and individuals. The detected number of mRNA and lncRNA genes was comparable between the five biofluids, ranging from 12 591 in plasma to 15 429 in amniotic fluid for mRNA, and from 4179 in plasma to 5764 in saliva for lncRNA (Figure 4A, Table S3). Most of mRNAs and lncRNAs were in low abundance. In plasma, only 4.9% of mRNA genes were with TPM >10, whereas in urine and seminal plasma, the rates were 24.2% and 25.5%. The lncRNAs with TPM > 10 were also with a low percentage, as the most of the biofluids were below 10% except for urine, the rate of which was 28%. The number of detected miRNA and piRNA was different among body fluids. Plasma has the most miRNA genes (980 on average) and least piRNA genes (670). Urine has the least miRNAs (335). Seminal plasma has the most piRNAs (2029). We also observed consistent result of tRNA in all biofluids (ranging from 411 to 420) (Figure S8). For miRNAs, the rate of miRNAs from plasma with RPM > 10 was 25.1%, and more than 60% in other biofluids.

Next, we identified differentially abundant genes among these body fluids (Table S5). According to the PCA results, samples of different biofluids could be classified by the profiles of differentially abundant cell‐free mRNA, lncRNA, miRNA and tRNA, respectively, except piRNA (Figures 4B and S9). Most differentially abundant genes had exclusively high abundance in their related biofluids (Figures 4C and S10). For example, *TRPM8*, which has been proved as a marker for high‐risk prostate cancer and was detected overrepresented in blood plasma,^49^ had a TPM of 0.065 in plasma and 30.0 in seminal plasma. The TPM of *UGT2B7*, a non‐coding RNAs marker of liver cancer,^50^ was 0.098 in plasma and 6.18 in urine. The TPM of the pre‐eclampsia marker *FABP1* ^51^ was 0.5 in plasma and 120 in amniotic fluids (Figure 4D).

We used PaGenBase and GO enrichment and KEGG pathway analyses^52^ to interpret the differentially abundant cf‐mRNA in different biofluids (Figure 4E,F). Differentially abundant cf‐mRNAs in blood plasma were annotated as tissue‐specific genes of bone marrow, spleen, thymus or lung and were functionally enriched in gas transport, lymphocyte activation, myeloid cell differentiation, adaptive immune response and leukocyte migration pathways, reflecting the origins of cfRNAs and the functions of originated tissues. Similarly, origin analysis and functional annotation of differentially abundant genes in urine, seminal plasma and amniotic fluid presented the features of their major contributing tissues, whereas immune pathways and blood‐specific genes were annotated for cf‐mRNAs in saliva, implying the contribution of immune cells. Then we applied CIBERSORT^8^ and xCell,^53^ two algorithms analysing the infiltration of immune cells, to characterize the profiles of biofluids’ cf‐mRNA. We calculated the absolute score, which scales the relative immune cell fractions to absolute abundance, and as expected, plasma contained the most amount of immune cell‐origin cfRNA (.460 ± .240). The saliva also had a relatively high absolute score, with significant difference to other biofluids (.204 ± .005, *p* < .05). The immune score, which is a composite score of immune cell types, was calculated by xCell. Similar to the absolute score, plasma and saliva had the relatively high immune score (.605 ± .199 and .773 ± .041), indicating the high immune cell rate in these two biofluids versus other biofluids (*p* < .001) (Figure 4G). Our results demonstrated that PALM‐Seq could profile cfRNAs from different biofluids and identify the markers with the information of tissue‐of‐origin.

### 3.7 Characterization of cfRNA fragments in different biofluids

**FIGURE 5:**
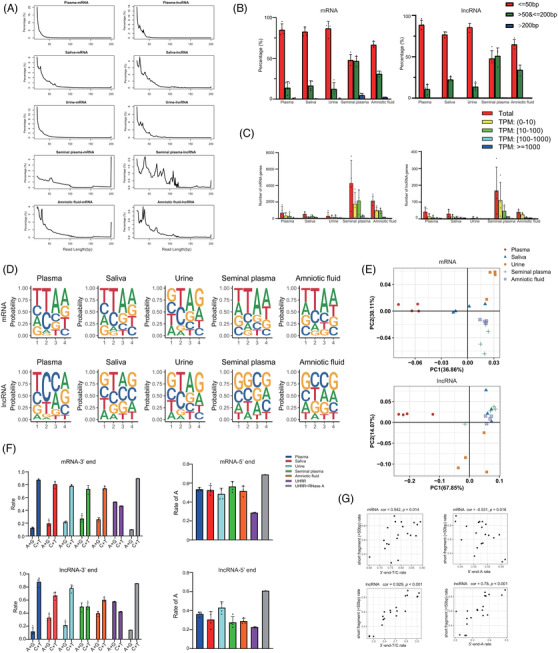
**Characterization of the cell‐free RNA (cfRNA) fragments in different biofluids**. (A) The distribution of mRNAs (left panel) and long non‐coding RNAs (lncRNAs) (right panel) fragment length in five biofluids using the paired‐end sequencing data. The *X*‐axis shows the read length (bp), whereas the *Y*‐axis shows the rate of each different size of sequence fragments. (B) The percentage of mRNA (left panel) and lncRNA (right panel) fragments with different lengths. The fragments are divided into <50 bp, 50–200 bp, >200 bp. (C) The number of mRNAs (left panel) and lncRNAs (right level) with long fragments (>200 bp fragments), divided by abundance value. (D) The frequency plot shows the frequency of each type of four nucleotides at the 4‐mer end motifs of mRNAs (upper panel) and lncRNAs (lower panel) in five body fluids. (E) Principal component analysis (PCA) plots based on the frequency of all 256 possible 4‐mer end motifs of mRNAs (upper panel) and lncRNAs (lower panel). Each point represents a single biofluid sample. (F) The ratio of pyrimidine and purine at the 3′ ends (left panel) and adenine at the 5′ end (right panel) of mRNAs and lncRNAs in different biofluids. Fractured RNAs of Universal Human RNA Reference (UHRR) by heat or RNase A treatment are used as control of intracellular RNAs. (G) Scatter plot shows the correlation between the rate of short fragments (<50 bp) and the ratio of pyrimidine and purine at the 3′ ends (left panel) and adenine at the 5′ end (right panel)

In addition to profiles of amount, we further analysed the length and cleavage sites of cfRNA fragments in different body fluids using paired‐end sequencing data. Although cellular mRNAs and lncRNAs are usually longer than 200 nt,^54^ we found that reads shorter than 50 bp took a large fraction of both cf‐mRNA and cf‐lncRNA in blood plasma (97.43% ± 1.96% and 97.21% ± 1.98%), saliva (82.94% ± 5.71% and 77.15% ± 3.15%), urine (86.98% ± 8.42% and 85.93% ± 4.40%) and amniotic fluid (66.85% ± 3.91% and 65.21% ± 5.59%) (Figure 5A,B). However, the fraction of such short reads was comparable to reads with length between 50 and 200 bp for both mRNAs (48.07% ± 6.87% vs. 47.00% ± 5.52%) and lncRNAs (48.17% ± 9.43% vs. 51.37% ± 9.30%) in seminal plasma. Only a small proportion of cf‐mRNA and cf‐lncRNA fragments was longer than 200 nt, with the highest percentage in seminal plasma (4.93% ± 1.90% and .45% ± .16%) (Figure 5B). More cf‐mRNA and cf‐lncRNA genes with long fragments (>200 nt) were detected in seminal plasma compared with other biofluids, and the numbers were highly variable among individuals (Figure 5C). Although the genes with long fragments had various abundance, compared with the result of all fragments (Figure 4A), long cfRNA fragments were prone to highly abundant genes.

Next, we characterized the features of 4‐mer end motifs of cfRNA fragments among body fluids to propose clues for the process of RNA fragmentation. The frequency of each 4‐mer end motif was calculated using cf‐mRNA and cf‐lncRNA reads (Tables S6 and S7), and we also evaluated the frequency of nucleotides on each site in all body fluids (Figure 5D). Samples from the same biofluid tended to cluster together in the PCA results, especially for plasma and urine (Figure 5E). In order to remove the influence of sequences from differentially abundant genes in biofluids, we analysed the features of genes with high abundance ranked in top 100 among all biofluids. We found that samples also tended to cluster together according to the biofluid type, indicating that the motifs of cleavage sites of cfRNA fragments could reflect signatures of various biofluids (Figure S11). We then evaluated the frequencies of 5′‐end and 3′‐end bases of cell‐free mRNA and lncRNA fragments and used UHRR as control of intact RNAs as UHRR was randomly fragmented by heat before PNKT. Unlike UHRR, the pyrimidine dominated at the 3′ ends of nearly all biofluids’ mRNA and lncRNA except lncRNA in seminal plasma. Adenine dominated at the 5′ ends of mRNAs in all biofluids especially seminal plasma, whereas its rate of lncRNA was slightly lower. To directly test if RNases cause the observed nucleotide biases, we treated UHRR with RNase A and found that it recapitulated the terminal nucleotide biases (Figure 5F). The preferences of nucleotides at fragments’ ends were different between circulating and intact RNA and varied among biofluids.

As 3′‐end nucleotides of cf‐mRNA and cf‐lncRNA in biofluids showed a consistent pattern of RNase A–mediated degradation, we further analysed the correlation between the end bases and fragment length (Figure 5G). Our results showed that for mRNA, the percentage of fragments shorter than 50 nt was positively correlated with the rate of pyrimidine at 3′ ends (correlation coefficient = .542, *p* < .05), whereas negatively correlated with the percentage of adenine base at 5′ ends (correlation coefficient = −.531, *p* < .05). For lncRNA, the percentage of fragments shorter than 50 nt was positively correlated with the percentage of pyrimidine at 3′ ends (correlation coefficient = .929, *p* < .001) and the percentage of adenine base at 5′ ends (correlation coefficient = .78, *p* < .001). These analyses provided a proof‐of‐concept that PALM‐Seq could quantify cell‐free mRNA, lncRNA and small RNAs simultaneously and characterize cell‐free mRNA and lncRNA fragments, providing a way to study the generation processes and functions of cfRNAs.

## 4 DISCUSSION

In this study, we developed a new library preparation strategy, PALM‐Seq, to quantify and characterize cfRNA in multiple human biofluids. PALM‐Seq simultaneously captures cell‐free mRNA, lncRNA and small RNAs regardless of length and efficiently depletes abundant rRNAs. The profiles and characterizations of blood plasma cfRNA by PALM‐Seq showed the contribution of placenta‐derived RNA, when comparing pregnant and non‐pregnant women. We also applied PALM‐Seq to describe the signatures of cfRNA in various biofluids, including the abundance features, fragment lengths, bases at fragmental ends and 4‐mer end motifs, which implied the promising applications of cfRNA investigation.

In order to profile and characterize cfRNA, we designed PALM‐Seq through several improvements. Treating cfRNA with T4 PNK could capture more RNA fragments.^20^, ^21^ Poly(A) tailing allows more efficient capture of scarce cfRNAs.^55^ RNase H and DNase I digestions allow the depletion of abundant rRNA and potentially contaminated DNA to improve sequencing efficiency and accuracy. Meanwhile, the direct addition of these asymmetrical adaptors to RNA molecules also made PALM‐Seq a strand‐specific RNA‐Seq method. The high correlation coefficient of UHRR or HBR standards expression profiles between PALM‐Seq and traditional methods demonstrated the quantitative accuracy of PALM‐Seq. Furthermore, PALM‐Seq captures the original RNA molecules, making it possible to describe cfRNA fragments.

The existing cfRNA sequencing strategies are focusing on either long or short RNA fragments, and it remains unclear which strategy is more representative. PALM‐Seq libraries were constructed regardless of RNA length and successfully reflected length distributions of cellular RNA biotypes, so we analysed the length of cf‐mRNA and cf‐lncRNA in biofluids. As our data show, cfRNAs were degraded to different extents in distinct biofluids. cfRNAs in saliva, urine and blood plasma were more degraded than seminal plasma,^6^, ^56^, ^57^, ^58^ showing larger fractions of short reads. More than 10 000 protein‐coding genes were detected in these body fluids by PALM‐Seq, whereas variable numbers of mRNAs were detected using TruSeq RNA exome library prep kit focusing on long RNAs, with 11 868 in seminal plasma and 2094 in urine.^48^ It implied that the detection of mRNA using long RNA‐Seq strategies tend to be affected by the degree of RNA degradation in biofluids. Taken together, the majority of cf‐mRNA and cf‐lncRNA fragments in biofluids are short, and PALM‐Seq captures RNA fragments regardless of length enabling efficient detection.

The small RNA‐Seq (NEBNext Small RNA Library Preparation Kit^59^ and TruSeq small RNA kit^20^) mainly detected miRNAs. PALM‐Seq with no_PNKT‐no_TD was similar to the small RNA‐Seq method, which mainly detected the small RNAs with 5′‐phosphate and 3′‐hydroxyl. The phospho‐RNA‐seq was designed to enrich both mRNA and miRNAs, whereas the problem is that the rRNAs make up a large proportion of their library. Using PALM‐Seq, the rates of mRNAs, lncRNAs and small RNAs were relatively high, along with the depletion of abundant RNAs.

One of the advantages of PALM‐Seq is that it could capture nearly all RNA biotypes in biofluids or cells and tissues. The simultaneous detection of different RNA biotypes could characterize physiological and pathological changes comprehensively and makes it possible to study the regulation networks of RNAs. Applying PALM‐Seq on plasma samples of COVID‐19 patients, researchers have found potential regulation network between *IL‐6R*, *miR‐451a* and three lncRNAs, providing a reasonable explanation to the elevated cytokine storms in COVID‐19 patients.^60^ PALM‐Seq could capture exogenous genes from pneumonia‐related microorganisms in the plasma of COVID‐19 patients, offering a method to screen the immune response and microbial infection at the same time. Another interesting finding was that the SARS‐CoV‐2‐biased codons in tRNA pools accumulated in the plasma of COVID‐19 patients, which may increase the replication of the virus.^61^ Using PALM‐Seq, researchers could monitor the health status on many common RNA biotypes.

cfRNA is actively or passively released from cells and could be degraded by various RNases in cells or biofluids, leading to short fragments. Because RNases have preferences for cleavage sites, we analysed 3′ end and 5′ end of cf‐mRNA and cf‐lncRNA fragments to observe potential footprints left by RNases. Compared to no_PNKT‐no_TD, treatment with PNK‐no_TD increased the number of detected mRNA and lncRNA (as shown in Table S3) and the fragments with 3′ pyrimidine at the same time (for mRNA, 56% in no_PNKT‐no_TD condition and 82% in PNKT‐no_TD condition), whereas treatment with TD did not increase the number of detected mRNA and lncRNA significantly (as shown in Table S3, PNKT‐no_TD vs. PNKT‐TD), nor the fragments with 3′ pyrimidine (for mRNA, 82% in PNKT‐no_TD condition and 85% in PNKT‐TD condition). These results indicated that PNKT was the main factor that captured most of the short fragments. This inference was also supported by the phospho‐RNA‐seq. Compared to no‐PNK treatment, PNK treatment could capture more mRNA and lncRNA genes and enriched the fragments with 3′ pyrimidine, which was in accordance with our results. Coincidently, RNase A family, a group of RNases commonly observed in biofluids, cleaves RNAs with preference at 3′ pyrimidine to generate 5′‐ hydroxyl and 3′‐phosphate products that could be captured by PNKT.^25^ Most cf‐mRNA and cf‐lncRNA fragments in biofluids showed higher rates of C and T bases at 3′ ends except cf‐lncRNA in seminal plasma, which was quite different from mRNA and lncRNA fragments in the heat‐fragmented UHRR libraries. The conclusion was further confirmed by the preference of 3′ pyrimidine and 5′ adenine of the mRNA and lncRNA fragments digested by RNase A in the UHRR libraries. Moreover, the 4‐mer end motifs of cf‐mRNA and cf‐lncRNA fragments could differentiate biofluids in the PCA analysis, proving that the cfRNA molecules carry the signatures of cfRNA processing. The differences of biofluid RNases in biotypes and concentration have been studied^62^ and may contribute to the molecular features of cfRNA fragments.

In‐line with our expectations, the rates of 3′‐end pyrimidine of cf‐mRNA and cf‐lncRNA and 5′‐end adenine of cf‐lncRNA were positively correlated with the rates of short fragments. cf‐mRNA and cf‐lncRNA in biofluids were mainly degraded by RNase A, leaving the 3′ C/T and 5′ A. The exception is that the rates of 5′‐end A were negatively related to the rates of cf‐mRNA short reads. One of the possible reasons is that these two types of RNAs undergo different RNA processing in cells and extracellular fluids, and lncRNA is generally less stable than mRNA.^63^, ^64^ However, the mechanisms underlying the genesis of these characters of cfRNAs are largely unknown and worth exploring. Our data demonstrated that cfRNA molecular features varied and could be characterized by PALM‐Seq, and the features inferred the differences between cf‐mRNA and cf‐lncRNA processing across biofluids.

The overall profiles and molecular features of cfRNA in blood plasma, saliva, urine, seminal plasma and amniotic fluid were first described by PALM‐Seq in this study and could distinguish the types of biofluids. The profiling of cf‐mRNA reflected the major tissues contributing to the biofluids and their biological functions. The immune score analysis presented that the fractions of immune cells were significantly higher in saliva and blood plasma than in the other three body fluids, which is in consistent with the observation that saliva contains a large number of granulocytes.^65^ In addition, placenta‐derived RNAs were successfully detected by PALM‐Seq in pregnant maternal blood plasma, showing the contributions of other tissues’ RNA besides blood cells. At present, blood plasma is a valuable resource for liquid biopsy, as it flows through the body, and has been widely used to identify cfRNA markers for multiple conditions. After we profiled cfRNA in different biofluids, a group of genes formerly identified signatures for diseases in blood plasma showed high abundance and functional relation in other fluids, such as *TRPM8*, *UGT2B7* and *FABP1*.^5^, ^49^, ^50^ The insights of tissue‐of‐origin derived from the RNA profiles in different biofluids could guide to select the most appropriate biofluids for liquid biopsy. Furthermore, the molecular features described by PALM‐Seq showed the potential ‘fragmentomics’ of cfRNA. The change of RNase activity was reported to be related to pathological states, including cancer^66^ and infection.^67^ We speculate that the molecular features of cfRNA may change in specific physiological or pathological status, providing promising biomarkers like cfDNA fragmentome.^27^ PALM‐Seq comprehensively characterized cfRNAs in biofluids and exhibited the huge potential of cfRNA in non‐invasive liquid biopsy.

Though PALM‐Seq showed good performance on cfRNA characterization, several modifications could be made to enhance its quantification abilities. First, to increase the recovery of the 5′‐capped mRNA or the aminoacyl‐tRNA, the treatment of mRNA decapping enzymes or peptidyl‐tRNA hydrolase before library preparation could be added.^68^, ^69^ We have tried to de‐cap the input cfRNAs using RppH (NEB, cat. M0356S) before constructing PALM‐Seq libraries. Our data showed no significant differences with or without de‐capping for cfRNA from plasma. It indicated that PALM‐Seq was compatible to the treatments of removing the modifications. Second, size selection could be applied to enrich the interested RNA biotypes.^20^ Third, the Unique Molecular Identifiers could be added to increase the accuracy of profiling of samples with extremely low concentration of cfRNAs.^70^ Forth, depletion probes could be adjusted for specific removal purposes. Thus, PALM‐Seq provides an ideal sequencing strategy for cfRNA investigation.

## 5 CONCLUSIONS

In this study, we developed a new cfRNA sequencing method named PALM‐Seq, which took advantage of T4 PNK and RNase H to quantify and characterize cfRNAs in different biofluids. Using PALM‐Seq, we could successfully identify differentially abundant mRNA, lncRNA and miRNA simultaneously from the blood plasma during pregnancy. We also characterized cfRNAs in different biofluids. The profiles of cf‐RNAs in different biofluids varied and reflected the function of originated tissues, and immune cells significantly contributed RNA to blood plasma and saliva. We also found that cfRNAs from different biofluids have different length and end motifs. The landscapes of biofluids’ cfRNAs generated by PALM‐Seq exhibit the potential capacity of cfRNA in liquid biopsy and imply the promising future of cfRNA application.

## FUNDING INFORMATION

This work was supported by The National Key Research and Development Program of China (Grant no. 2018YFC1004900); The Science, Technology and Innovation Commission of Shenzhen Municipality (Grant nos. JCYJ20170412152854656, JCYJ20180703093402288); The Shenzhen Peacock Plan (Grant No. KQTD20150330171505310).

## CONFLICT OF INTEREST

The authors declare that they have no competing interests.

## Supporting information

Supp. Figures Information

Supp. Table S1 Information

Supp. Table S2 Information

Supp. Table S3 Information

Supp. Table S4 Information

Supp. Table S5 Information

Supp. Table S6 Information

Supp. Table S7 Information
