# Supplementary material for: Polyadenylation ligation‐mediated sequencing (PALM‐Seq) characterizes cell‐free coding and non‐coding RNAs in human biofluids
Source: Clin Transl Med. 2022 Jul 20;12(7):e987. doi: 10.1002/ctm2.987 (PMC9299576; doi:10.1002/ctm2.987)
Supplement: Supplementary file 1 — Supp. Figures Information [file CTM2-12-e987-s005.pdf]

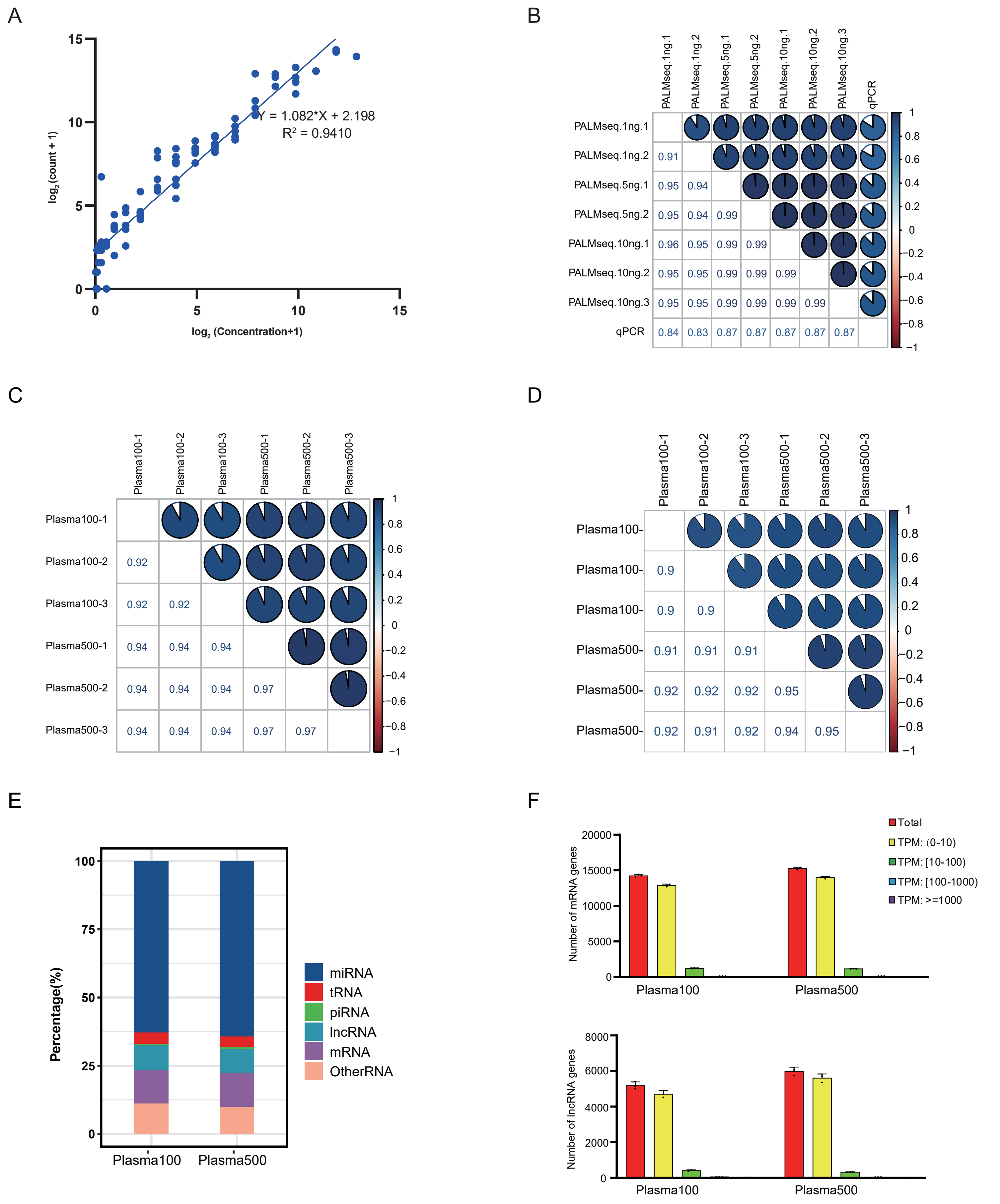

Figure S1. Evaluation of PALM-Seq in RNA standards. A, Correlation between read counts and expected concentration of ERCC RNAs. The correlation coefficient is calculated using Spearman correlation. **B**, Correlation analysis (R^2 value) of mRNAs between PALM-Seq with the input from 1 ng to 10 ng and the qPCR of UHRR. The correlation coefficient is calculated using Pearson correlation. **C-D**, The Correlation coefficient of (**C**) mRNAs and (**D**) lncRNAs in PALM-Seq libraries with the input cfRNAs from 100 μL and 500 μL pooled plasma. **E**, The proportion of different RNA biotypes in libraries from 100 μL and 500 μL of pooled plasma. **F**, The number of mRNAs (upper) and lncRNAs (lower) are shown as mean ± S.E.M. (n = 3). Three repeats are conducted for each input volume. Related to Figure 2.

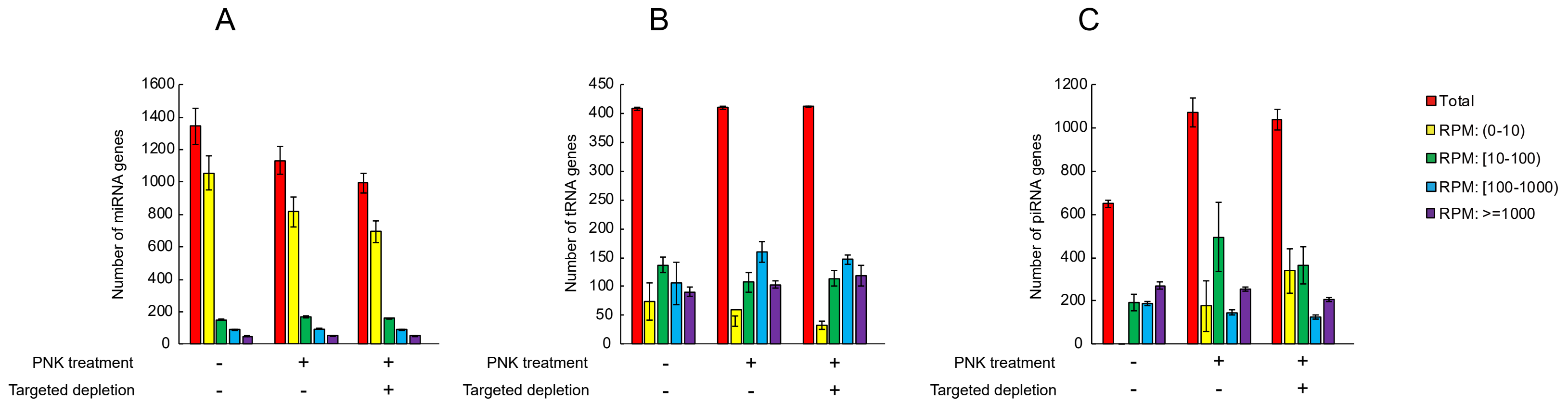

Figure S2. Number of small RNAs detected by different treatments of PALM-Seq.
A, miRNAs; **B**, tRNAs; **C**, piRNAs. Related to Figure 2.

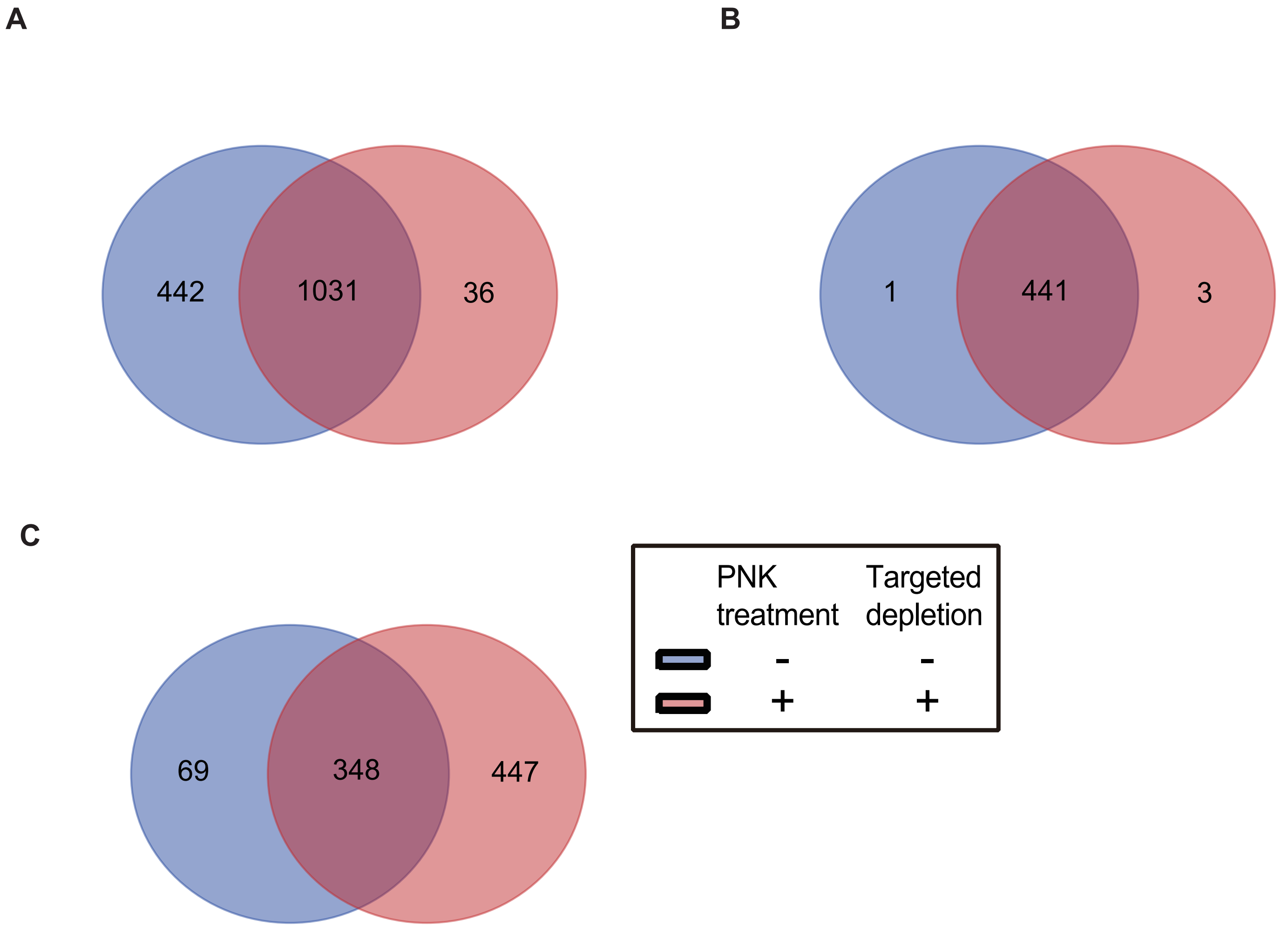

Figure S3. Differentially detected RNA biotypes shown by Venn diagrams with different treatments. A, miRNAs; **B**, tRNAs; **C**, piRNAs. The RNAs that were detected in two or more samples were counted. Related to Figure 2.

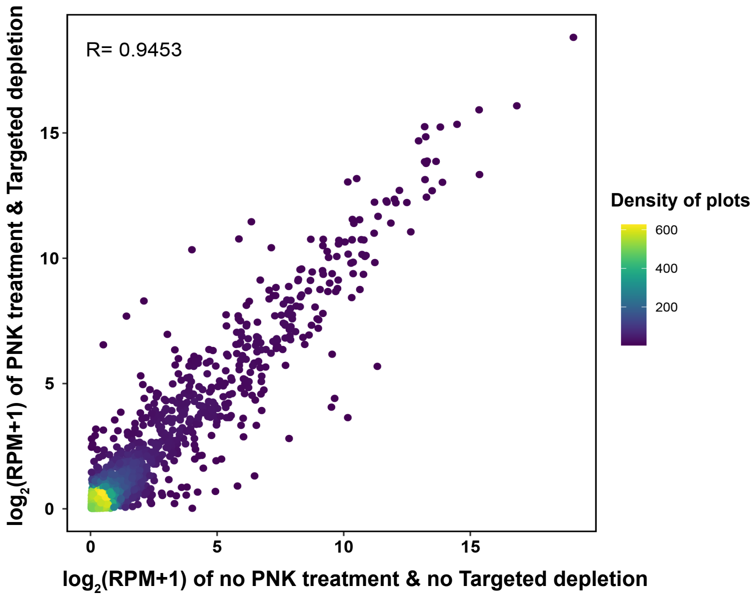

Figure S4. The influence of PNK treatment & Targeted depletion on detected miRNA profile. The Pearson correlation is calculated through simple linear regression of log_2(RPM+1) for miRNA. Related to Figure 2.

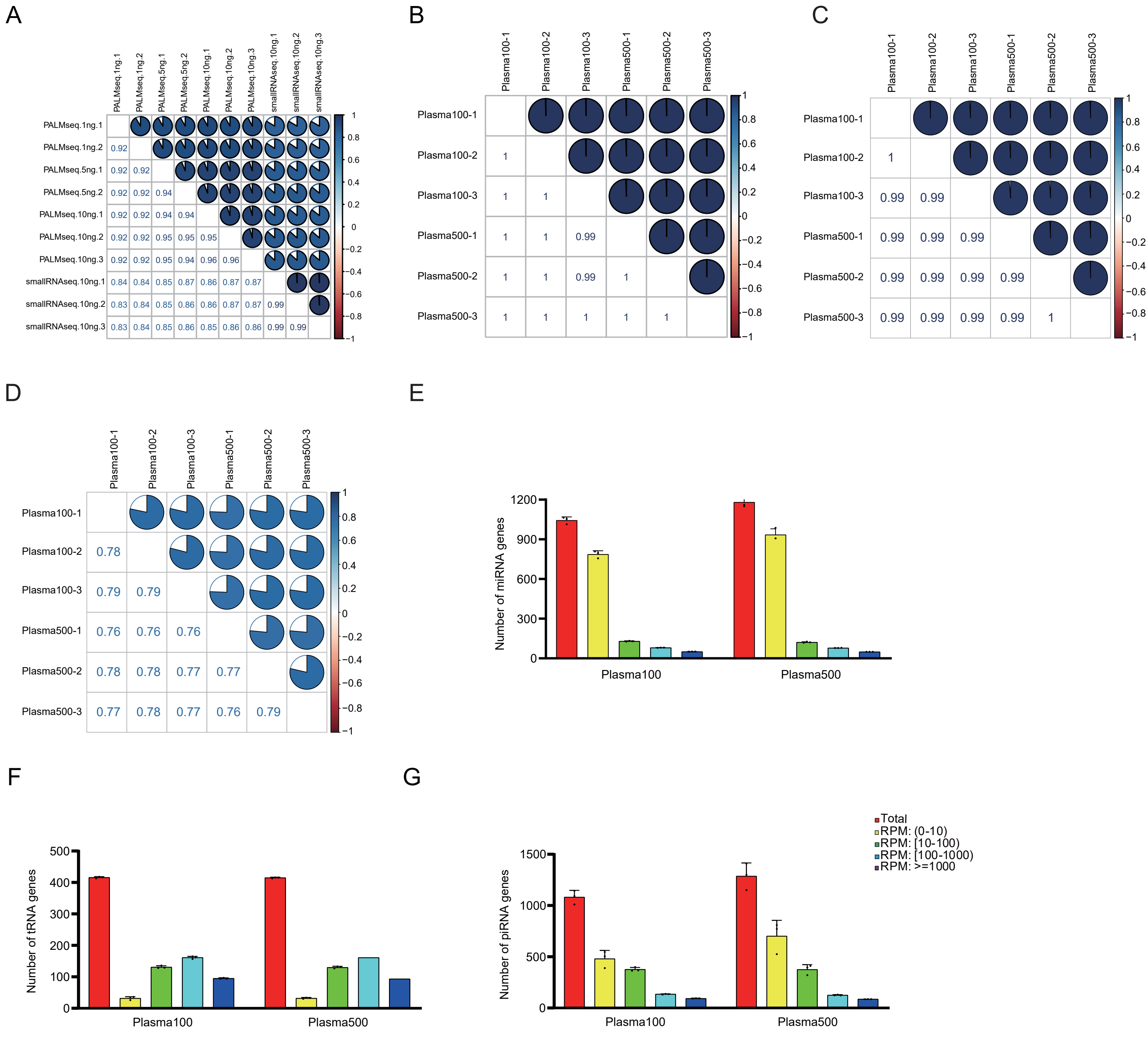

Figure S5. Performance of PALM-Seq in small RNA quantification. A, Correlation analysis (R^2 value) of miRNAs between PALM-Seq with the input from 1 ng to 10 ng and the small RNA seq of HBR. The correlation coefficient is calculated using Pearson correlation. **B-D,** The Correlation coefficient of (**B**) miRNAs, (**C**) tRNAs and (**D**) piRNAs from PALM-Seq libraries with the input cfRNAs from 100 μL and 500 μL pooled plasma. **E-G**, The number of (**E**) miRNAs, (**F**) tRNAs and (**G**) piRNAs are shown as mean ± S.E.M. (n = 3). Three repeats are conducted for each input volume. Related to Figure 2.

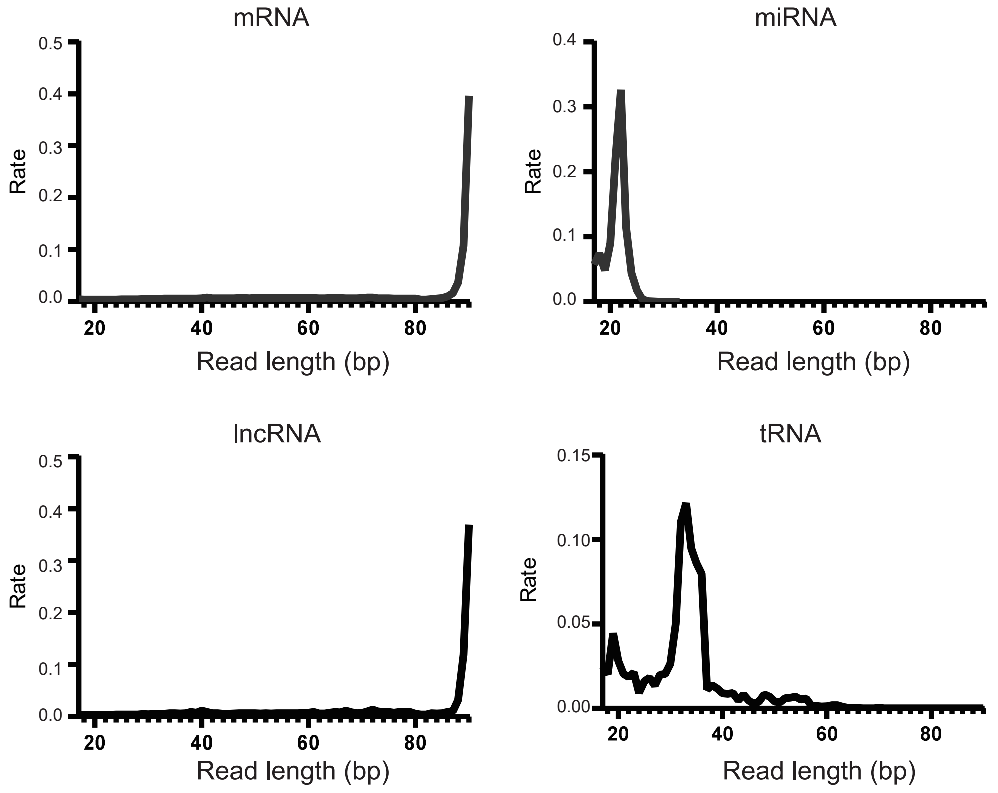

Figure S6. Length distribution of fragments in libraries of UHRR constructed by PALM-Seq. The X-axis shows the different read lengths (bp), while the Y-axis shows the rate of each different size of sequence fragments. Related to Figure 2.

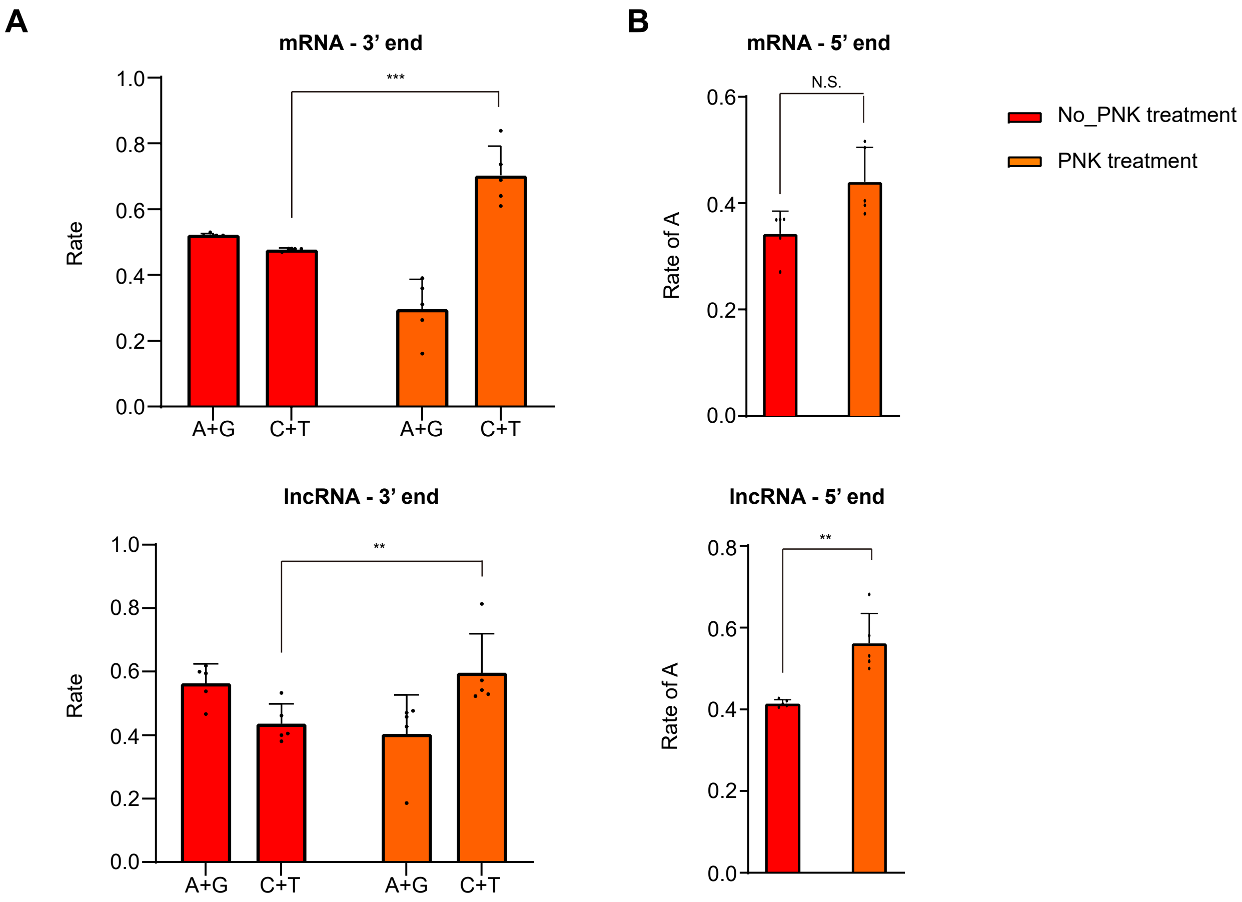

Figure S7. The rate of nucleotides at the 3’- and 5’- ends of fragments in phospho-RNA-seq libraries. A, The rate of pyrimidine and purine at the 3’- end of mRNA (upper panel) and lncRNA (lower panel). B, The rate of adenine at 5’-end of mRNA (upper panel) and lncRNA (lower panel). Related to Figure 2.

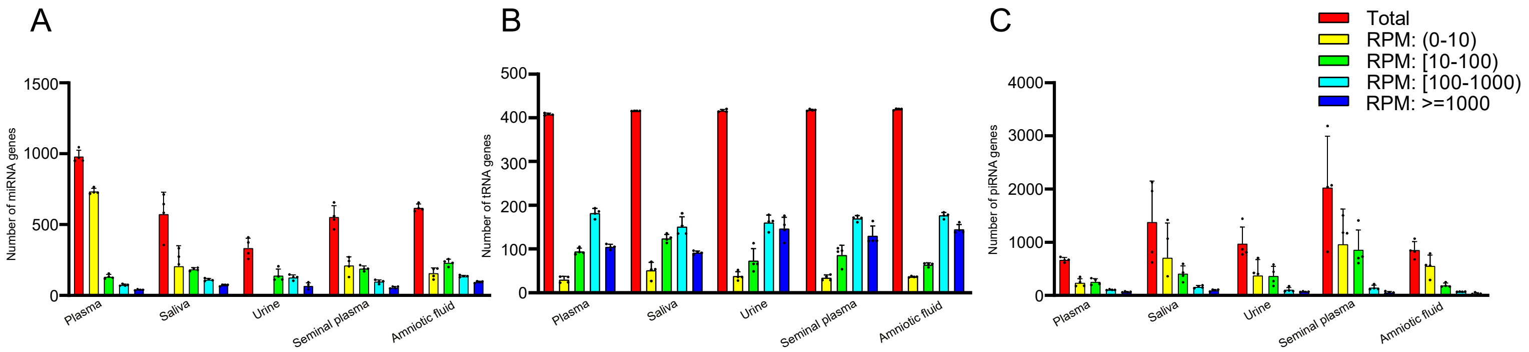

Figure S8. Detection of small RNAs in different biofluids. A-C, The number of (**A**) miRNAs, (**B**) tRNAs and (**C**) piRNAs detected in different biofluids. Four samples are used for each biofluid type. Related to Figure 4.

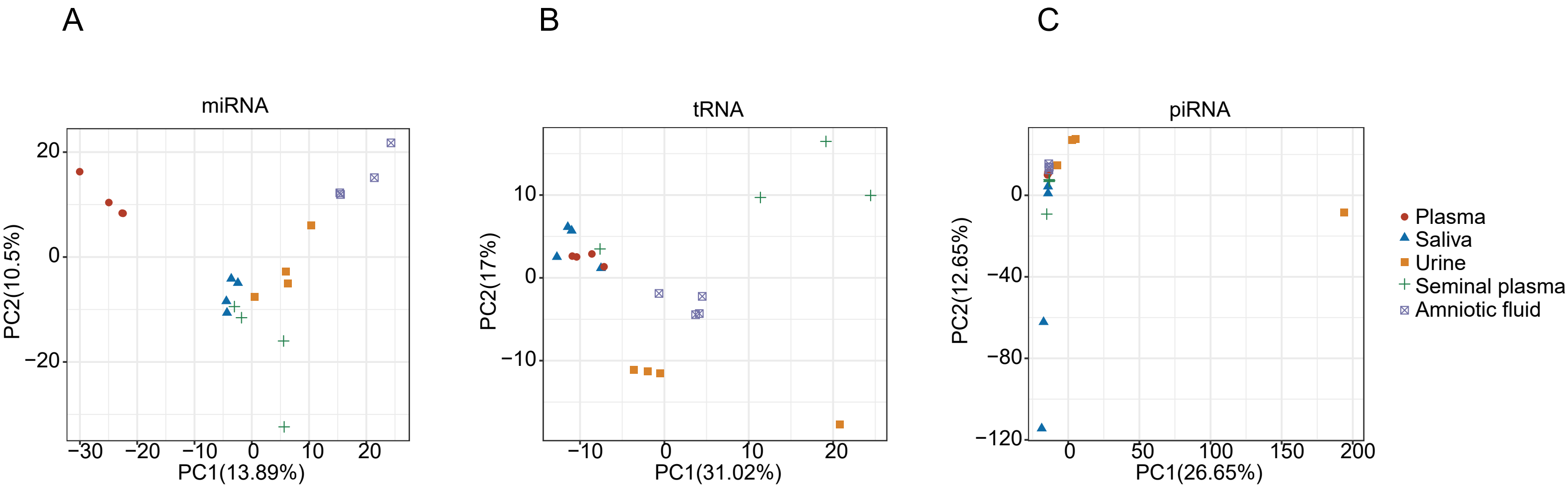

Figure S9. PCA plots generated using (A) miRNAs, (B) tRNAs and (C) piRNAs RPM.
Each point represents a single biofluid sample. Related to Figure 4.

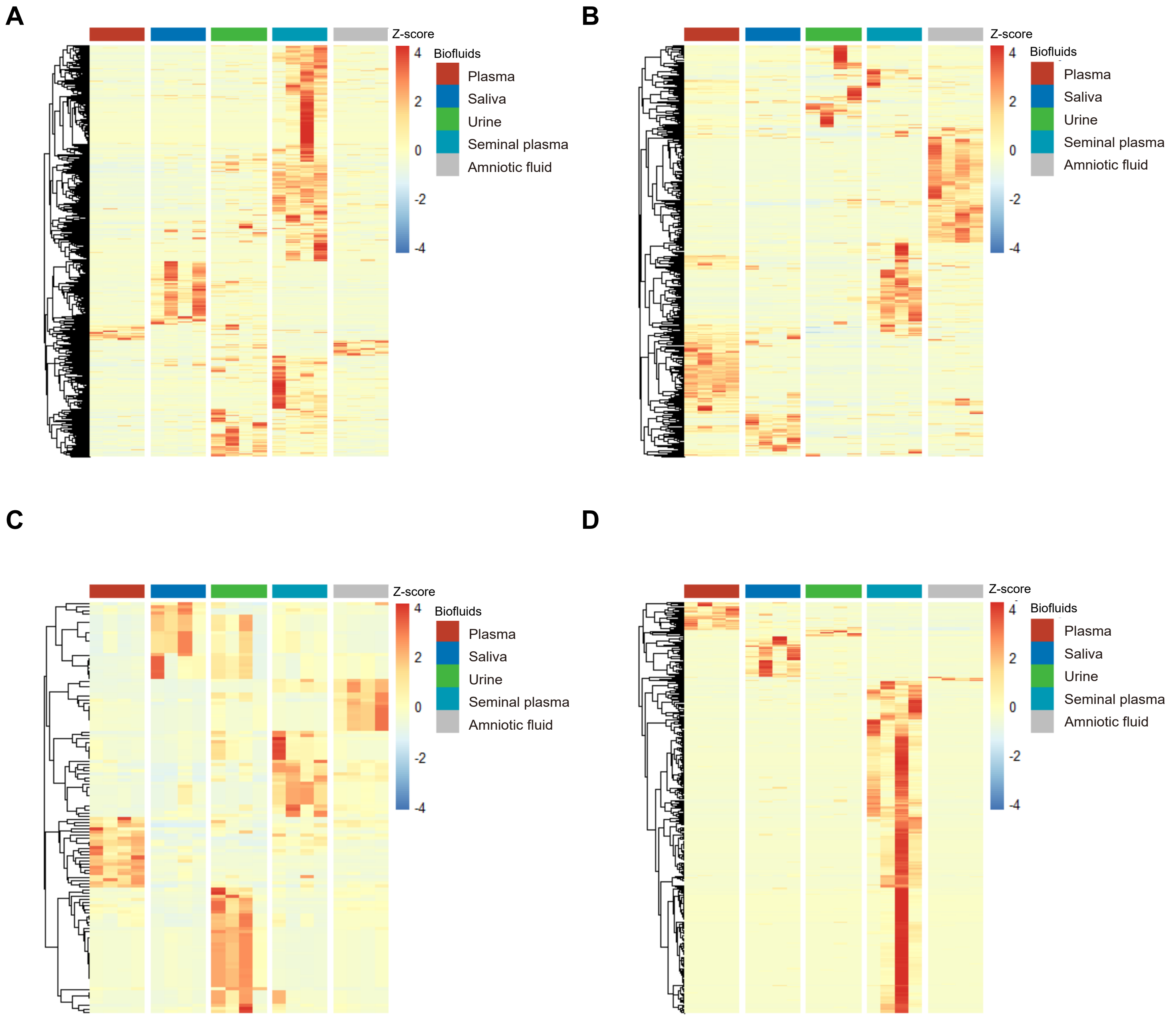

Figure S10. Heatmap of differentially abundant RNA in five biofluids. The heatmaps depict biofluid-specific (**A**) lncRNA, (**B**) miRNA, (**C**) tRNA and (**D**) piRNA. Related to Figure 4.

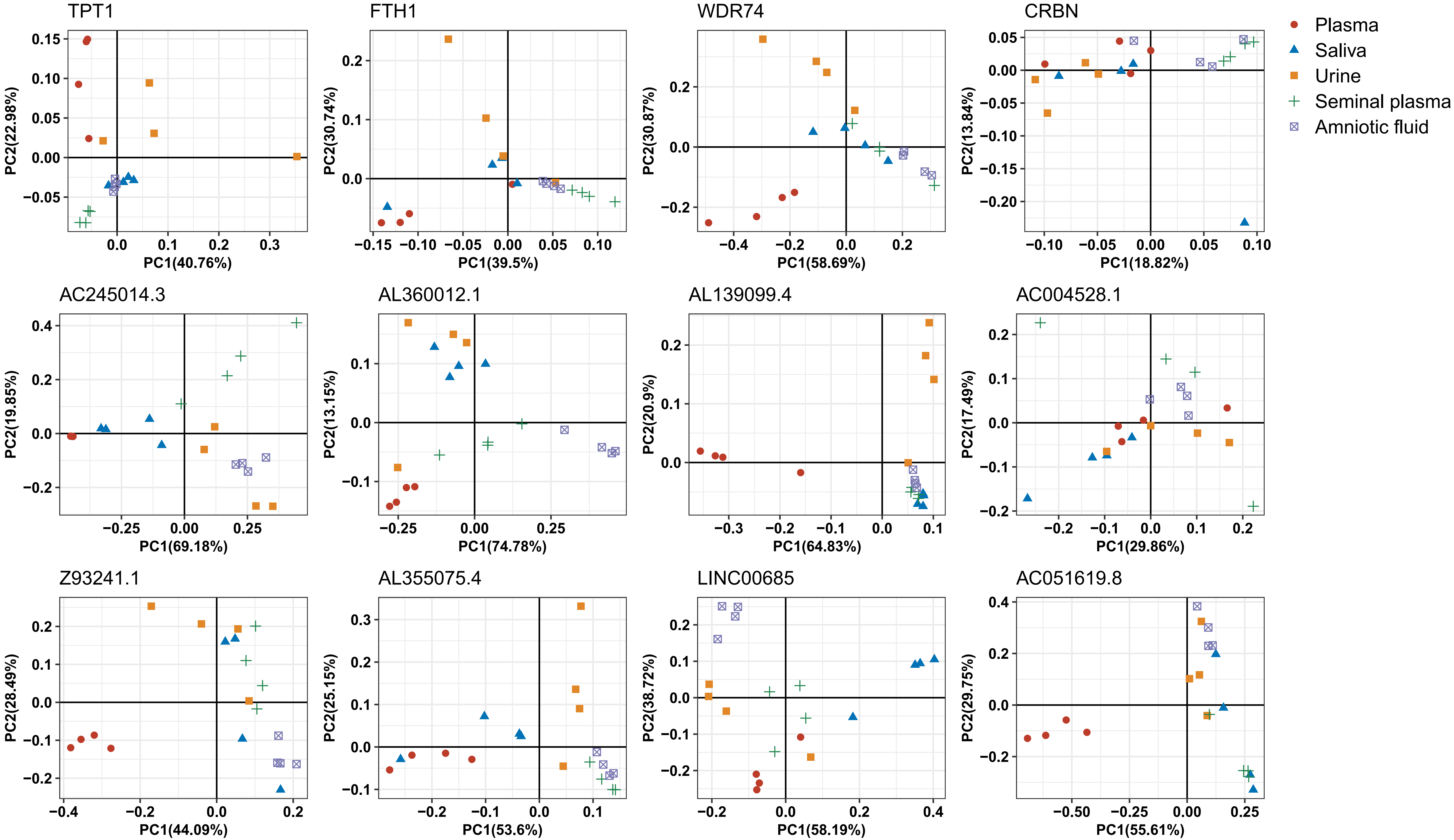

Figure S11. The PCA plots based on the frequency of end motifs of mRNA or lncRNA fragments with high abundance in all biofluids. Related to Figure 5.
