# Supplementary material for: Polyadenylation ligation‐mediated sequencing (PALM‐Seq) characterizes cell‐free coding and non‐coding RNAs in human biofluids
Source: Clin Transl Med. 2022 Jul 20;12(7):e987. doi: 10.1002/ctm2.987 (PMC9299576; doi:10.1002/ctm2.987)
Supplement: Supplementary file 3 — Supp. Table S2 Information [file CTM2-12-e987-s004.pdf]

Table S2. The oligos and primers used in this study

Depletion Oligo for Y RNA and Vault RNA

Target	Oligo Sequence
RNY4	GCCAGTCAAATTTAGCAGTGGGGGGTTGTATACCAACTTTAGTGACACT
RNY4	TGACACTAATGTTAATAAGTTCTGATAACCCACTACCATCGGACCAGCC
RNY1	GACTAGTCAAGTGCAGTAGTGAGAAGGGGGGAAAGAGTAGAACAAGGAG
RNY1	GAAAGAGTAGAACAAGGAGTTCGATCTGTAACTGACTGTGAACAATCAA
RNY1	ACTGACTGTGAACAATCAATTGAGATAACTCACTACCTTCGGACCAGCC
RNY3	GGCTAGTCAAGTGAAGCAGTGGGAGTGGAGAAGGAACAAAGAAATCTGT
RNY3	AACTGGTTGTGATCAATTAGTTGTAAACACCACTGCACTCGGACCAGCC
RNY5	CAGCAAGCTAGTCAAGCGCGGTTGTGGGGGGAGACAATGTTAAATCAAC
RNY5	AGACAATGTTAAATCAACTTAACAATAACCCACAACACTCGGACCAACT
VTRNA	GGACTGGAGAGCGCCCGCGGGTCTCGAACAACCCAGACAGGTTGCTTGT
VTRNA	TTGTTTCAATTAAAGAACTGTCGAAGTAACCGCTGAGCTAAAGCCAGCC

Primers for Library Preparation and RT-qPCR

Primer	Oligo Sequence
5’ adaptor	GAACGACAUGGCUACGAUCCGACUUNNNN (RNA)
RT primer	GACCGCTTGGCCTCCGACTTTTTTTTTTTTTTTTTTTTTTTTVN
Seq PCR Primer-F	GAACGACATGGCTACGATCCGACTT (5’ -Phosphorylation)
Seq PCR Primer-R	TGTGAGCCAAGGAGTTG[barcode]TTGTCTTCCTAAGACCGCTTGGCCTCC
RT pimer (qPCR)	GACCACGCGTATCGATGTCGACTTTTTTTTTTTTTTTTVN
miRNA out Primer	GACCACGCGTATCGATGTCGAC
B2M-F	CCGTGTGAACCATGTGACTT
B2M-R	GATGCTGCTTACATGTCTCG
CGA-F	CAGAGTCCACTTGCTGTGTA
CGA-R	GCCGTGTGGTTCTCCACTTT
CSH1-F	CAGCTCACCTAGTGGCAATG
CSH1-R	GACGGCACCAGCCTCTTGAA
CSH2-F	ATTCCTGCATGACTCCCAGA
CSH2-R	ATGTTGGAGGGTGTCGGAAT
PLAC4-F	AAGTGCCTTCCCAAGTCTCA
PLAC4-R	GACAGTTGTCAAAGGTCCTAG
S100A8-F	GGAGTTCCTCATTCTGGTGA
S100A8-R	CTCAGCTACTCTTTGTGGCT
RN7SL	CCTGTGAATAGCCACTGCACT
mir-526b-5p	CTTGAGGGAAGCACTTTCTG
mir-519d-3p	AAGTGCCTCCCTTTAGAGTG
mir-515-5p	TTCTCCAAAAGAAAGCACTTTCTG
mir-512-3p	AGTGCTGTCATAGCTGAGGT
mir-454-3p	GTGCAATATTGCTTATAGGG
